# Title, Table of Contents and Acknowledgements

**DOI:** 10.1080/26410397.2026.2646529

**Published:** 2026-07-24


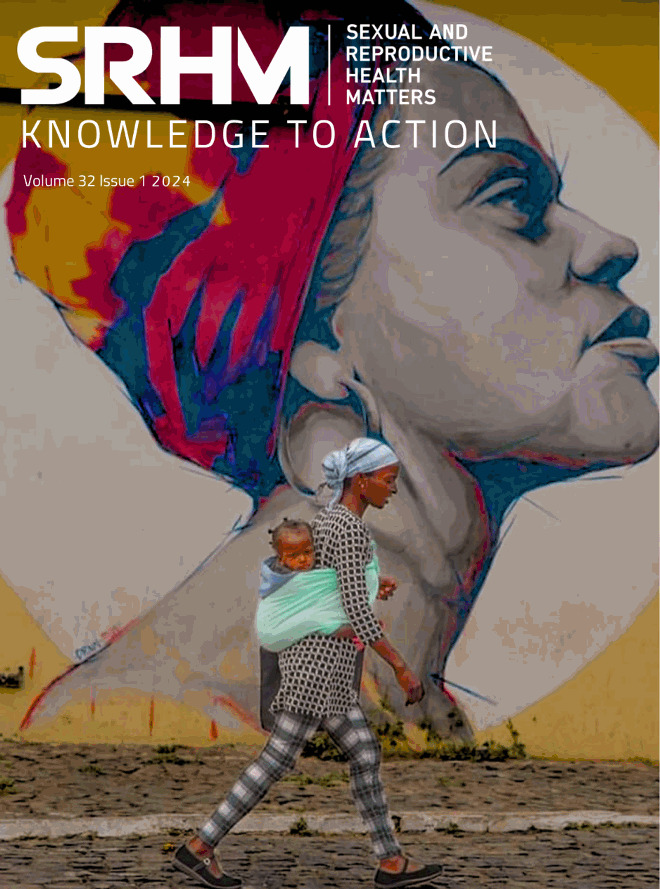


**Table:** 

**Editorials**	
*Eszter Kismödi, Emma Pitchforth, TK Sundari Ravindran, Laura Ferguson, Mindy Jane Roseman, Jane Cottingham, Sapna Desai*	The continuing fight for abortion rights: taking stock of the evidence
*Laura Ferguson, Sapna Desai*	Sexual and reproductive health and rights in Palestine – securing spaces to speak out
*Anne Philpott, Paromita Vohra*	Finding the cosmos of intimacies: where pleasurable safe sex dances with liberation
**Commentaries**	
*Angela Baschieri, Chiagozie Udeh*	International Conference on Population and Development (ICPD) Programme of Action and Climate Action: an intersecting agenda
*Susana T Fried, Alice M Miller, Rupsa Mallik, Ivana Radačić, Esteban Restrepo-Saldarriaga*	“The (mis)use of evidence in contested rights: commentary on the UN Special Rapporteur on violence against women and girls’ report on “prostitution and violence”
*Katrina Karkazis, Michele Krech*	What do oral contraceptive pills have to do with human rights abuses in sport?
*Evelyne Opondo, Jade Maina, Nelly Munyasia*	Lessons from Kenya on sexual reproductive health and rights policy making: the need to centre voices from Africa in global discourses
*Alicia Ely Yamin, Lisa Cosgrove*	Extending the concept of “obstetric violence” to post-partum experiences: cautions regarding the “first ever” pill for post-partum depression
**Reviews**	
*Eva Åkerman, Anna Wängborg, Maria Persson, Marie Klingberg-Allvin*	Experiences of menstrual health in the Nordic countries: a scoping review of qualitative research, applying an intersectional lens
*Shatha Elnakib, Ahmed K. Ali, Kate Mieth, Venkatraman Chandra-Mouli*	Mapping the evidence on interventions that mitigate the health, educational, social and economic impacts of child marriage and address the needs of child brides: a systematic scoping review
**Research articles**	** **
*Ahmed K. Ali, Alka Barua, Rajesh Mehta, Venkatraman Chandra Mouli*	Nimble adaptations to sexual and reproductive health service provision to adolescents and young people in the early phase of the COVID-19 pandemic
*Karin Båge, Anna Kågesten, Olalekan Uthman, Mariano Salazar, Bi Puranen, Signe Svallfors, Anna Mia Ekström, Helena Litorp*	Attitudes toward sexual and reproductive health and rights and their associations with reproductive agency: a population-based cross-sectional study in Ethiopia, Kenya, and Zimbabwe
*Radilaite Cammock, Tengihia Pousini, Malcolm Andrews*	Reflections on using Talanoa methodology to engage with Pacific youth in Aotearoa New Zealand about their sexual and reproductive health
*Suzanne Day, Asia Carter, Anna Lloyd, Arlene C. Seña, Justin D. Radolf, Joseph D. Tucker*	Barriers and facilitators of participation in syphilis vaccine trials: a qualitative analysis to inform trial design and community engagement in the United States
*Cristina Espinosa da Silva, Margarita Santibanez, Adrienne R.S. Lee, Lorena S. Pacheco, Stephanie Brodine, Miguel A. Fraga, Taylor B. Desmarais, Noe C. Crespo, Javier Martínez Hernandez, Marianne McKennett, Richard S. Garfein*	Sexual and reproductive health awareness and practices among adolescents and adults in a rural farming community in Baja California, Mexico: a quantitative and qualitative cross-sectional study
*Meredith Evans, Alexandra Rego, Nkem Ogbonna, Kate Welsh, Sidrah K. Zafar, Lucy C. Barker, Anne Berndl, Janice Du Mont, Yona Lunsky, Amy McPherson, Lesley A. Tarasoff, Ashley Vandermorris, Hilary K. Brown*	Impacts of the COVID-19 pandemic on access to sexual and reproductive health services for women and transgender people with disabilities in Canada: a qualitative study
*Laura Ferguson, Sarah Emoto, Sofia Gruskin*	Laws governing access to sexual health services and information: contents, protections and restrictions
*Serah Gitome, Petina Musara, Miria Chitukuta, Felix Mhlanga, Bismark Mateveke, Thandiwe Chirenda, Nyaradzo Mgodi, Prisca Mutero, Allen Matubu, Gift Chareka, Charles Chasakara, Caroline Murombedzi, Tinei Makurumure, Carolyn Smith-Hughes, Elizabeth Bukusi, Craig R. Cohen, Stephen Shiboski, Lynae Darbes, George W. Rutherford, Z. Michael Chirenje, Joelle M. Brown*	“First was to sit down and bring our minds together”. A qualitative study on safer conception decision-making among HIV sero-different couples in Zimbabwe
*Lucia Guerra-Reyes, Rossmary D. Márquez-Lameda, Ruhun Wasata, and Oakley Byrne*	Provider perspectives on maternal care challenges for Black and Latine Women in Indiana: a qualitative interview study
	
*Emma Halper, Blake Erhardt-Ohren, Melissa Cobb, Oscar Hidalgo-Mora, Sebastián Ospina-Henao, Amari O’Bannon, Roger Rochat, Subasri Narasimhan, Anna Newton-Levinson*	Socio-ecological influences on access to abortion care in Costa Rica: a qualitative analysis of key perspectives from clinical and policy stakeholders
*Mary Higgins, Sharon Cooley, Deirdre Hayes-Ryan, Brendan Dempsey*	Approaches to a crisis in early pregnancy: an explorative qualitative study of medical students and doctors in training in Ireland, using a story completion model
*Manali Karmakar*	Reproductive trauma, vulnerable mothers, and disenfranchised grief: reflecting on the affective dimensions of surrogacy practice in Indian literary and film narratives
*Ijeoma Opara, Emmanuella Asabor, Jaleah Rutledge, Jasmin R. Brooks Stephens, Sandy Cayo, Beatriz Duran-Becerra, Jasmine Abrams*	Empowerment in prevention: a qualitative inquiry into Black girl-centred strategies for reducing HIV/STI and drug misuse risk
*Elizabeth Pleasants, Karen Weidert, Lindsay Parham, Emma Anderson, Eliza Dolgins, Coye Cheshire, Cassondra Marshall, Ndola Prata, Ushma Upadhyay*	Abortion access barriers shared in “r/abortion” after Roe: A qualitative analysis of Reddit community post-Dobbs decision leak in 2022
*Josefina Pruneda Paz, Andrea García-Egea, Constanza Jacques-Aviñó, Ana Maria Besoaín Cornejo, Laura Medina-Perucha*	An intersectional approach on menstrual inequity as lived by women in circumstances of socioeconomic vulnerability in an urban and rural setting in Spain: a qualitative study
*Lore Remmerie, Guncha Annageldiyeva, Kayleigh Grossman, Caesar Kaba Kogoziga, Nicole Leonetti, Ana Mosiashvili, Shreya Shrestha, Tisungane Sitima, Evi Stuckens, Michael Tetteh Doku, Aslan Temirkhanov, Diana Marcela Zambrano, Heidi Mertes, Kristien Michielsen*	Towards an inclusive and culturally sensitive conceptualisation of sexual well-being of young people: preliminary framework development using a modified Delphi methodology
*María Félix Rodríguez-Camacho, María José Sanchís-Ramón, Gaby Ortiz Barreda, Diana Gil-González*	Service providers’ perspectives and reproductive (in)justice among Roma women: a qualitative study in Spain
*María Félix Rodríguez-Camacho, María José Sanchís-Ramón, Gaby Ortiz Barreda, Diana Gil-González*	Service providers’ perspectives and reproductive (in)justice among Roma women: a qualitative study in Spain
*Andrea Whittaker, Trudie Gerrits, Karin Hammarberg, Lenore Manderson*	Access to assisted reproductive technologies in sub-Saharan Africa: fertility professionals’ views
**Bookshelf**	
*Chen Jung*	Siegl, Veronika. (2023). Intimate Strangers: Commercial Surrogacy in Russia and Ukraine and the Making of Truth. Cornell University Press

**Table:** 

**Executive Editor:** Emma Pitchforth **Chief Executive**: Eszter Kismödi **Senior Editor:** Sarah Keogh, TK Sundari Ravindran **Managing Editor:** Pete Chapman **Monitoring Editor**: Pathika Martin **Communications Manager**: Jessica MacKinnon **Operations Manager**: Amy Guthrie **Associate Editors:** Laura Ferguson, Atsumi Hirose, Ramya Kumar, Helen Potts, Mindy Jane Roseman, Nina Sun **Translation:** Françoise de Luca-Lacoste translated abstracts from English to French and Lisette Silva translated abstracts from English to Spanish. **Cover image:** *Unattributed photograph (source: Instagram / @iamcvbp)*	**Funding:** We thank the Open Society Foundations for their generous support to SRHM's work in 2024. **Copyright © 2026 Sexual and Reproductive Health Matters.** This is an Open Access journal distributed under the terms of the Creative Commons Attribution License (http://creativecommons.org/licenses/by/4.0/), which allows for sharing and adapting the work for any purpose, even commercially, provided appropriate credit is given with a link to the originally published item, a reference to the author(s) and links to their homepages, reference to the license under which the article is published and a link to this, as well as an indication of any changes that have been made to the original. ISSN (Online) 2641-0397

**Peer reviewers:**Gillian Abel, Laura Adair, Yemi Adewoyin, Taofeekat Oluwatosin Adigun, Joshua Amo-Adjei, Nada Amroussia, Mariana Prandini Assis, Karan Babbar, Chiara Bercu, Irina Bergenfeld, Jane Bertrand, Deevia Bhana, Emily R Boniface, Jonna Both, Doortje Braeken-van Schaik, Caila Brander, Anna Brittain, Julie Buser, Sara Causevic, Elizabeth Charron, Pooja Chitneni, Kathryn Church, Ernestina Coast, Tamaryn L Crankshaw, Anna Kheyfets Daoud, Natasha Davidson, Farah Diaz-Tello, Alison Drake, Niklas Envali, Sarah Emoto, Sam Everingham, Dabney P Evans, Eva Fiks, John Flores, Jane Freedman, Christine Galavotti, Dibyasree Ganguly, Jagadishwor Ghimire, Lynda Gilby, Lesley Gittings. Camila Giugliani, Piedad Gómez-Torres, Anna Gordon, Karen Trister Grace, Lorraine Grimes, Candice Groenewald, Laura K Grubb, Firoza Haffejee, Aisha Hutchinson, Mobolaji Ibitoye, William Joe, Anna Kågesten, Nathalie Kapp, Jane Kelly, Courtney Kerestes, Katherine Kissler, Nagendra Kumar, Sara Rivenes Lafontan, Christina Laurenzi, Ophra Leyser-Whalen, Mengjia Liang, Tambudzai M. Manjonjo, Karen Maxwell, Yunia Mayanja, Gitau Mburu, Desirée Mena-Tudela, Alice Miller, Kathryn Mishkin, Priyadarshini Mishra, Lorretta Favour Ntoimo, Mary O Obiyan, Lina Papadopoulou, Lydia H Pecker, Sachini Perera, Kate Pincock, Eliã Pinheiro Botelho, Monika Pobiruchin, Carrie Purcell, John Reynolds-Wright, Damien W Riggs, Sally B Rose, Keith Sabin, Marta Schaaf, Clémence Schantz, Nancy Marie Sidun, Ridwan Islam Sifat, Rachael Sorcher, Silpa Srinivasulu, Shunji Suzuki, Dina Taha, Tigest Tamrat, Kun Tang, Laura Tarzia, Vincent J Tukei, Thilde Vildekilde, Jens Walldorf, Yuanyuan Wang, Drew A Westmoreland, Christina Zampas, Kornelia Zareba

www.srhm.org/www.srhmjournal.org

@SRHMJournal

